# Kynurenic Acid and Its Synthetic Derivatives Protect Against Sepsis-Associated Neutrophil Activation and Brain Mitochondrial Dysfunction in Rats

**DOI:** 10.3389/fimmu.2021.717157

**Published:** 2021-08-12

**Authors:** Marietta Z. Poles, Anna Nászai, Levente Gulácsi, Bálint L. Czakó, Krisztián G. Gál, Romy J. Glenz, Dishana Dookhun, Attila Rutai, Szabolcs P. Tallósy, Andrea Szabó, Bálint Lőrinczi, István Szatmári, Ferenc Fülöp, László Vécsei, Mihály Boros, László Juhász, József Kaszaki

**Affiliations:** ^1^Institute of Surgical Research, Faculty of Medicine, University of Szeged, Szeged, Hungary; ^2^Institute of Pharmaceutical Chemistry and Research Group for Stereochemistry, Hungarian Academy of Sciences, University of Szeged, Szeged, Hungary; ^3^Department of Neurology, Interdisciplinary Excellence Centre, Faculty of Medicine, University of Szeged, Szeged, Hungary; ^4^Neuroscience Research Group, Hungarian Academy of Sciences (MTA)-University of Szeged (SZTE), Szeged, Hungary

**Keywords:** N-methyl-D-aspartate receptor, blood-brain barrier, mitochondrial respiration, brain injury, neutrophil extracellular trap

## Abstract

**Background and Aims:**

The systemic host response in sepsis is frequently accompanied by central nervous system (CNS) dysfunction. Evidence suggests that excessive formation of neutrophil extracellular traps (NETs) can increase the permeability of the blood–brain barrier (BBB) and that the evolving mitochondrial damage may contribute to the pathogenesis of sepsis-associated encephalopathy. Kynurenic acid (KYNA), a metabolite of tryptophan catabolism, exerts pleiotropic cell-protective effects under pro-inflammatory conditions. Our aim was to investigate whether exogenous KYNA or its synthetic analogues SZR-72 and SZR-104 affect BBB permeability secondary to NET formation and influence cerebral mitochondrial disturbances in a clinically relevant rodent model of intraabdominal sepsis.

**Methods:**

Sprague–Dawley rats were subjected to fecal peritonitis (0.6 g kg^-1^ ip) or a sham operation. Septic animals were treated with saline or KYNA, SZR-72 or SZR-104 (160 µmol kg^-1^ each ip) 16h and 22h after induction. Invasive monitoring was performed on anesthetized animals to evaluate respiratory, cardiovascular, renal, hepatic and metabolic parameters to calculate rat organ failure assessment (ROFA) scores. NET components (citrullinated histone H3 (CitH3); myeloperoxidase (MPO)) and the NET inducer IL-1β, as well as IL-6 and a brain injury marker (S100B) were detected from plasma samples. After 24h, leukocyte infiltration (tissue MPO) and mitochondrial complex I- and II-linked (CI–CII) oxidative phosphorylation (OXPHOS) were evaluated. In a separate series, Evans Blue extravasation and the edema index were used to assess BBB permeability in the same regions.

**Results:**

Sepsis was characterized by significantly elevated ROFA scores, while the increased BBB permeability and plasma S100B levels demonstrated brain damage. Plasma levels of CitH3, MPO and IL-1β were elevated in sepsis but were ameliorated by KYNA and its synthetic analogues. The sepsis-induced deterioration in tissue CI–CII-linked OXPHOS and BBB parameters as well as the increase in tissue MPO content were positively affected by KYNA/KYNA analogues.

**Conclusion:**

This study is the first to report that KYNA and KYNA analogues are potential neuroprotective agents in experimental sepsis. The proposed mechanistic steps involve reduced peripheral NET formation, lowered BBB permeability changes and alleviation of mitochondrial dysfunction in the CNS.

## Introduction

Sepsis is defined as a dysregulated host response to infection, which can lead to life-threatening organ failure (1). The brain is among the potentially injured vital organs; and central nervous system (CNS) abnormalities assessed by sequential organ failure assessment (SOFA) scores can be present in up to 70% of patients, in association with higher mortality (2, 3).

Although the pathomechanism of sepsis-associated encephalopathy is not fully understood, it is recognized that the CNS responds to peripheral cytokine release through an increase in blood–brain barrier (BBB) permeability (4). BBB leakage associated with edematous cerebral cortex lesions (5–7) and injury of hippocampal-cerebellar structures have already been identified at the early phase (~6–8h) of various models of sepsis (8). In parallel with damage to BBB integrity, infiltration of activated polymorphonuclear leukocytes into brain tissues also occurs (9). The activation of circulatory leukocytes with neutrophil extracellular trap (NET) formation leads to excessive release of proteases and generation of reactive oxygen species (ROS), which exacerbates BBB damage (10–12). NETs are web-like DNA and intracellular protein structures with constant components, such as histones, myeloperoxidase (MPO) and neutrophil elastase, while other components depend on stimuli, e.g., pathogens, cytokines, antibodies and immune complexes (13). Most importantly, a regulated form of neutrophil cell death with NET formation defined as NETosis correlates with the severity of organ failure (14).

Along with BBB injury and immune activation, cerebral mitochondria are also affected soon after an inflammatory insult (~12–24h) at least in rodent experiments. Functional and morphological changes within the organelles and changes in microglial energy metabolism (15) have been demonstrated, manifested by decreased respiratory chain function and oxidative phosphorylation (OXPHOS) (16) and loss of mitochondrial membrane potential (ΔΨmt) (17). These events may ultimately lead to a release of mitochondrial damage-associated molecular patterns to the extracellular space, which further stimulate the immune response. In addition, mitochondria-driven inflammation may contribute to cell death, multiorgan failure or long-term cognitive dysfunction as well (18–20).

In this context, it has been shown that the neuronal N-methyl-D-aspartate receptors (NMDA-Rs) can play an important role in sepsis-induced neuroinflammation and sensory dysfunction, but the underlying molecular mechanisms remain unknown (21, 22). Kynurenic acid (KYNA), a metabolite of the tryptophan–L-kynurenine pathway, is a naturally occurring antagonist of NMDA-R and acts as an endogenous neuroprotectant in a number of brain diseases (23). Furthermore, exogenously administered KYNA exerted cell-protective effects in neuronal (23) and non-neuronal tissues (e.g., liver and intestine) (24) in various pro-inflammatory circumstances. In addition to glutamate receptor antagonism, KYNA acts as an agonist for G protein-coupled receptor GPR35 and the aryl hydrocarbon receptor and regulates glutamatergic neurotransmission and immune activation (25). Previously, we have already shown that KYNA and its synthetic analogue, termed SZR-72, ameliorated sepsis induced mitochondrial dysfunction (decreased oxygen consumption and ΔΨmt) in the rat liver (17). Nevertheless, the mitochondrial effects of these compounds in the CNS have not been examined before.

Given this background, the main goal of our study was to characterize the mechanism of a KYNA-based therapy specifically targeted for brain neuroprotection during a septic reaction. We hypothesized that exogenously administered KYNA and its BBB-permeable synthetic analogues (SZR-72 and SZR-104) might be therapeutic tools to reduce mitochondrial disturbances in the CNS by influencing peripheral NET formation and BBB permeability in a clinically relevant rodent model of intraabdominal sepsis.

## Materials And Methods

### Animals

The experiments were performed on male Sprague–Dawley rats (n_∑_=77; 410 ± 30 g) housed in plastic cages (21–23°C) with a 12/12h dark/light cycle and access to standard rodent food and water *ad libitum*. The study was performed in accordance with the National Institutes of Health guidelines on the handling and care of experimental animals and EU Directive 2010/63 for the protection of animals used for scientific purposes, and it was approved by the National Scientific Ethical Committee on Animal Experimentation under license number V/175/2018.

### Sepsis Induction and Treatments

The animals were randomly divided into sham-operated (n_∑_=15) and septic groups (n_∑_=62). Polymicrobial sepsis was induced with intraperitoneally (ip)-administered fecal inoculum, as described before (17, 26). Briefly, fresh feces samples were randomly collected from healthy rats (n=4–5), suspended and incubated in physiological saline (37°C, 5h). After filtering, the count for colony forming units (CFUs) in the suspension used for sepsis induction was determined with the standard poured plate count method. This analysis retrospectively demonstrated that the CFU range of the inducer inoculum was 1.02×10^6^–5.6 ×10^6^ CFU mL^-1^. Rats in the sham-operated groups received the same amount of saline ip. The septic animals were further divided into saline-treated (n=17), KYNA- (Sigma-Aldrich Inc., St. Louis, MO, USA; 160 μmol kg^−1^ ip; n=15), SZR-72- [N-(2-(dimethylamino)ethyl)-4-hydroxyquinoline-2-carboxamide hydrochloride, 160 μmol kg^−1^ ip; n=15] or SZR-104- [N-(2-(dimethylamino)ethyl)-3-(morpholinomethyl)-4-hydroxyquinoline-2-carboxamide, 160 μmol kg^−1^ ip; n=15] treated groups. Both SZR-72 and SZR-104 were synthesized by the Institute of Pharmaceutical Chemistry, University of Szeged, Hungary (27, 28). The compounds can be classified into acid (KYNA), amide (SZR-72) and aminoalkylated amide (SZR-104) derivatives. All bear the crucial 4-hydroxyquinoline-2-carboxyl scaffold (i.e. KYNA); however, one or two tertiary nitrogen bearing groups have been built-in. Thus, SZR-72 contains one cationic center, while SZR-104 contains two cationic centers, which has been previously proved to be responsible for better BBB-penetration (28) and could result in different biological effects. Treatments were performed in two steps (80 µmol kg^-1^; in 3 mL kg^-1^ saline each; pH=7.2–7.4) 16h and 22h after sepsis induction.

### Monitoring of Animal Well-Being

The general condition of the animals was evaluated at 6h and 16h after the induction of sepsis using a modified 0–9-point rat-specific sickness (RSS) scoring system, where a cumulative value above 6 was considered a humane endpoint for euthanasia (**Supplementary Table S1**). At the time of the RSS assessments, the animals received 10 mL kg^-1^ crystalloid solution subcutaneously (sc) (Ringerfundin, B. Braun, Hungary) to avoid dehydration and 15 µg kg^-1^ buprenorphine sc (Bupaq, Merck, USA) to maintain analgesia according to the Minimum Quality Threshold in Preclinical Sepsis Studies (MQTiPSS) recommendations (29).

### Experimental Protocol

**Figure 1:**
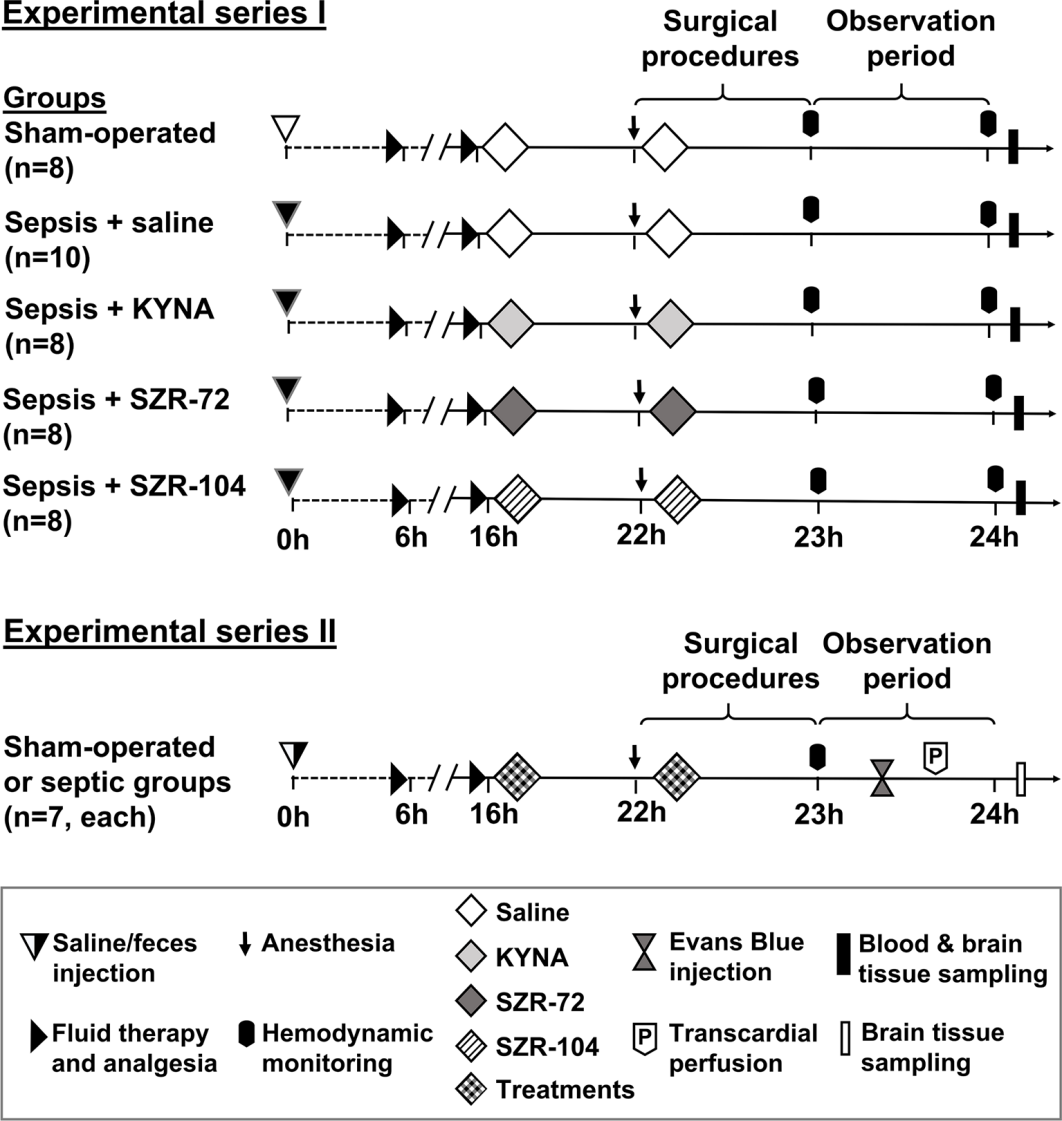
The schemes for the experimental protocols (groups, interventions and assessments). In Series I, sampling for NETosis parameters and measurements of brain mitochondrial oxygen consumption were conducted, whereas blood–brain barrier permeability measurements were performed in Series II. The animals were randomly assigned to sham-operated or septic groups, which later was further divided into four independent groups according to the treatment (saline, KYNA, SZR-72 or SZR-104) applied at the 16^th^ and 22^nd^ hours of sepsis.

The experiments were performed in two series (**Figure 1**). In Experimental Series I, sepsis-induced pathological changes in NETosis were estimated, and cerebral mitochondrial respiration analyzed. A second series of experiments had to be established to measure the BBB permeability of the cerebellum and hippocampus using the fluorescence Evans Blue technique to avoid methodology interference with the fluorescent dye (see later).

### Experimental Series I – Assessment of NETosis and Mitochondrial Functions

#### Surgical Interventions and Sampling

At 22h of sepsis, the animals were anesthetized ip with a mixture of ketamine (45.7 mg kg^−1^) and xylazine (9.12 mg kg^−1^). The rats were placed on a heating pad to maintain normal core body temperature (37°C). After tracheostomy, mechanical ventilation (Inspira Advanced Safety Ventilator 55-7058; Harvard Apparatus Inc., Holliston, MA, USA) was started with 7–8 mL kg^-1^ volume of room air. The ventilation parameters (tidal volume and breath rate) were set up based on arterial blood gas values (see later). PE50 tubing was placed into the right jugular vein to administer fluid infusion (10 mL kg^-1^ h^-1^ Ringerfundin iv) and to maintain anesthesia (ketamine 12 mg kg^-1^ h^-1^, xylazine 2.4 mg kg^-1^ h^-1^ and diazepam 0.576 mg kg^-1^ h^-1^ iv). The left carotid artery was also cannulated for continuous monitoring of the heart rate (HR) and the mean arterial pressure (MAP; SPEL Advanced Cardiosys 1.4; Experimetria Ltd., Budapest, Hungary). After the 30-min stabilization period, lactate levels of the venous blood were measured (Accutrend Plus Kit; Roche Diagnostics Ltd., Rotkreuz, Switzerland) to determine metabolic imbalance. MAP and HR data were recorded, and arterial blood samples were collected for blood gas analysis (Cobas b123; Roche Ltd., Basel, Switzerland). After 60 min of monitoring, HR and MAP values were registered, and arterial and venous blood samples were collected for blood gas analysis. Based on a standard formula (SaO_2_–SvO_2_)/SaO_2_), simplified oxygen extraction (OER) was calculated from arterial (SaO_2_) and venous oxygen saturations (SvO_2_). Lung function was determined by calculating the PaO_2_/FiO_2_ ratio (Carrico index) from partial arterial oxygen pressure (PaO_2_) and fraction of inspired oxygen (FiO_2_), which was 0.21.

Following a median laparotomy, blood samples were collected from the inferior vena cava into pre-cooled EDTA-coated tubes, centrifuged (1200 g at 4°C for 10 min) and stored at -70°C for later analysis. The rats were then sacrificed under deep anesthesia, followed by a quick decapitation. After the removal of the skin and skull bones, the hippocampus and cerebellum were dissected for analysis of mitochondrial respiratory functions and tissue MPO determination (**Figure 1**).

### Experimental Series II – Measurement of Blood–Brain Barrier Permeability

In the second series, sepsis induction, treatments with KYNA or KYNA analogues and the surgical preparations were identical with Experimental Series I (sham-operated n=7; saline-treated sepsis n=7; KYNA- n=7; SZR-72 n=7 and SZR-104-treated sepsis n=7, respectively). Following the hemodynamic and arterial blood gas measurement, Evans Blue (EB) dye was injected iv for the determination of BBB permeability as described earlier (30) (**Figure 1**).

Briefly, 2% EB (1 mL kg^-1^ dissolved in saline; Sigma-Aldrich Inc.) iv bolus was injected 23h after sepsis induction. After a 20-min circulation of the tracer, animals were perfused transcardially with 250 mL of saline to remove dye with a 8 mL min^-1^ flow rate until the liver was cleared and a colorless washing fluid appeared from the right atrium. After decapitation, the hippocampus and cerebellum were dissected and wet weight measurements were performed to calculate wet tissue/body weight ratio. Cerebral tissues were homogenized in trichloroacetic acid (50%, Sigma-Aldrich Inc.) to extract the dye from the tissue. After centrifugation (10,000 g, 4°C, 20 min), supernatant was collected and diluted 3:1 with ethanol (70%, Sigma-Aldrich Inc.). Fluorescence intensity was determined at a wavelength of 680 nm (RF-6000 Spectrofluorometer, Shimadzu Corporation, Kyoto, Japan), and tissue EB content was quantified from a standard linear curve derived from known amounts of dye and expressed in ng g^-1^ tissue.

### Rat Organ Failure Assessment

Rat organ failure assessment scores (ROFA) (17) were calculated based on the idea of adapting the SOFA scoring system to rats. The components were scored between 0 and 4 based on threshold values determined earlier. Lung function was assessed by calculating the Carrico index (PaO_2_/FiO_2_ ratio). Cardiovascular function was evaluated from MAP values registered during the monitoring phase. Metabolic imbalance caused by tissue hypoxia was indicated by blood lactate levels. Severity of kidney injury was determined from plasma urea levels, whereas liver dysfunction was assessed by measuring plasma alanine aminotransferase (ALT) levels using a Roche/Hitachi 917 analyzer (F. Hoffmann–La Roche AG, Switzerland). Rats with ROFA scores above 2 were considered as septic (**Table 1**).

**Table 1:** Threshold values of the components of the rat-specific organ failure assessment (ROFA) scoring system. Sepsis was defined as cumulative ROFA score above 2.

Score	ROFA parameters				
	Respiratory system	Cardiovascular system	Metabolism	Renal function	Liver function
	**PaO_2_/FiO_2_ ratio**	**MAP (mmHg)**	**blood lactate (mmol L^-1^)**	**plasma urea (mmol L^-1^)**	**plasma ALT (U L^-1^)**
**0**	400<	75<	<1.64	<7.5	<17.5
**1**	300–400	65–75	1.64–3	7.5–21	17.5–30.2
**2**	200–300	55–65	3–4	21<	30.2<
**3**	100–200	<55	4–5	–	–
**4**	–	–	5<	–	–

### Measurement of Cerebral Mitochondrial Functions

Mitochondrial oxygen consumption was assessed from the cerebellum and hippocampus using High-Resolution FluoRespirometry (17) (Oxygraph-2k; Oroboros Instruments, Innsbruck, Austria). In brief, the whole brain was removed rapidly, and the cerebellum and both the left and right hippocampi were dissected on a pre-cooled Petri dish. The remaining blood was washed out with ice-cold phosphate-buffered saline (pH 7.4). Tissue samples were weighed on an analytical balance (Precisia100A-300M; Precisa Gravimetrics AG, Dietikon, Switzerland), cut into small pieces (~10–15 mg) with a sharp scissors and then homogenized in five times the amount of Mir05 media (pH 7.1) using a Potter–Elvehjem homogenizer. All measurements were performed under continuous stirring (750 rpm) at 37°C in a 2 mL Mir05 respiration medium. After a stable baseline respiration had been achieved, LEAK respirations were evaluated after the oxidation of complex specific substrates: 10 mM glutamate, 2 mM malate (complex I-linked respirations; LEAK_GM_) or 10 mM succinate (complex II-linked respirations; LEAKs). Maximal capacities of oxidative phosphorylation (OXPHOS I and OXPHOS II) were achieved by saturating concentration of ADP (2.5 mM). Reverse electron transport-derived ROS production was inhibited with rotenone (complex I inhibitor; 0.5 μM), prior to the addition of succinate. The cytochrome c test (cytc; 10 μM) was used after the addition of ADP to assess the integrity of the outer membrane. ATP synthase was inhibited by oligomycin (2.5 μM) to evaluate LEAK respiration in a non-phosphorylating state (LEAK_Omy_). Electron transport-independent respiration (or residual oxygen consumption; ROX) was determined following complex III inhibition with antimycin A (2.5 μM). The respiratory control ratio (RCR), an index of coupling between respiration and phosphorylation, was expressed as a ratio of OXPHOS to LEAK_Omy_ state. The DatLab software (Oroboros Instruments, Innsbruck, Austria) was used for online display, respirometry data acquisition and analysis. Mitochondrial oxygen consumption was normalized to wet weight (cerebellum: 19 mg and hippocampus: 25 mg) and expressed in pmol s^−1^ mL^−1^.

### Measurements of Inflammatory Markers and Indices of NET Formation

#### Tissue and Plasma Myeloperoxidase

Circulating MPO level was regarded as an indicator of systemic neutrophil activation and NET formation, whereas MPO being retrieved from tissues (31) was regarded as a marker of neutrophil granulocyte infiltration in brain tissue. Plasma MPO level was detected from 100 µL undiluted plasma, while the tissue MPO content was measured after a 2-step extraction method from the pellet of the cerebral homogenate. The latter data were referred to the protein content of the sample and were given in mU mg protein^-1^.

#### Plasma Levels of Interleukin 6, Interleukin 1β, Citrullinated Histone H3, and S100B

Sandwich enzyme-linked immunosorbent assay (ELISA) kits were used to quantify proinflammatory cytokine interleukin 6 (IL-6; BioLegend, San Diego, CA, USA), NETosis inducer interleukin 1β (IL-1β; R&D Systems, Inc., Minneapolis, MN, USA), NETosis-related biomarker citrullinated histone H3 (Cit H3; MyBioSource, Inc., San Diego, CA, USA) and brain/BBB injury marker S100B (MyBioSource, Inc., San Diego, CA, USA). All measurements of plasma samples were carried out according to the manufacturers’ instructions.

### Statistical Analysis

The sample size estimation was based on a power analysis using the PS Power and Sample Size Calculation software (version 3.1.2). Data analysis was performed using a statistical software package (SigmaStat for Windows; Jandel Scientific, Erkrath, Germany). Normality of data distribution was analyzed with the Shapiro–Wilk test. Differences between groups were calculated by either one-way analysis of variance (ANOVA) completed with the Holm–Sidak post-hoc test or Kruskal–Wallis one-way ANOVA on ranks followed by Dunn’s method depending on the distribution of the data. Median values and 75^th^ and 25^th^ percentiles are provided in the figures; *P*<0.05 were considered significant.

## Results

### Characterization of Sepsis Progression

#### Changes in Oxygen Dynamics, Inflammatory Marker, and Organ Dysfunction Score

The overall health condition of the septic animals in all the groups deteriorated significantly to the same extent 16h after sepsis induction, as shown by the RSS scores (**Supplementary Figure S1**).

**Figure 2:**
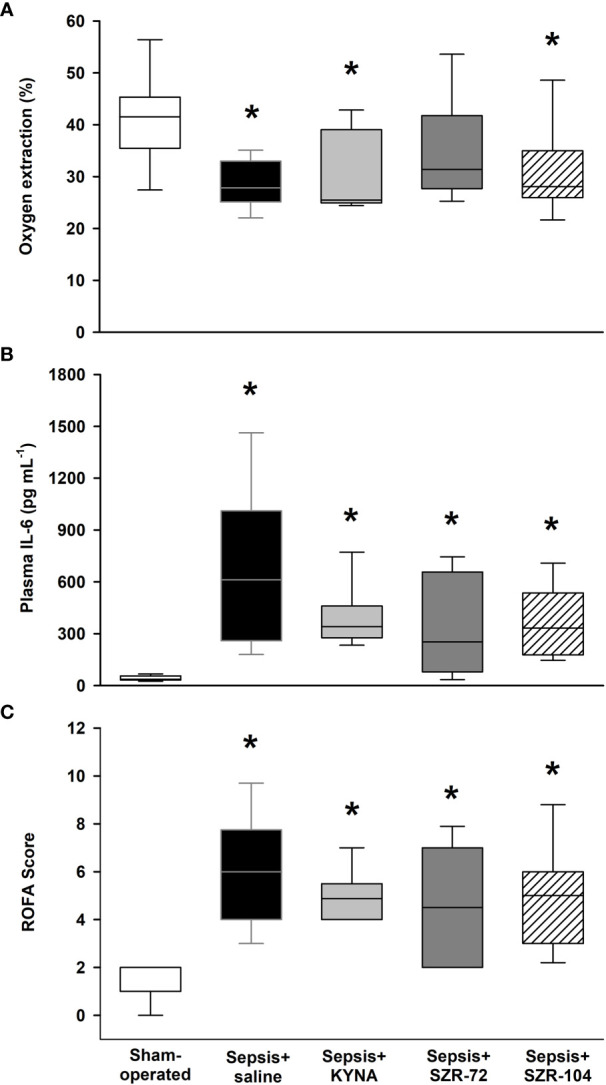
Oxygen extraction ratio (OER, **A**), plasma IL-6 levels **(B)** and rat-specific organ failure assessment (ROFA) score **(C)** in the sham-operated group and in the different sepsis groups treated with saline, KYNA, SZR-72 and SZR-104. The plots demonstrate the median (horizontal line in the box) and the 25^th^ (lower whisker) and 75^th^ (upper whisker) percentiles. Kruskal–Wallis test and Dunn’s *post-hoc* test; *P < 0.05 *vs*. sham-operated group.

When compared to the sham-operated animals, the saline-treated septic animals showed lower OER, but higher IL-6 and organ dysfunction (ROFA) scores 24h after sepsis induction (**Figures 2A–C**). The OER values in the SZR-72-treated septic animals, however, did not significantly differ from those of the sham-operated animals (**Figure 2A**). IL-6 and ROFA score elevations similar to those in the non-treated septic group were also evident in the septic groups treated with KYNA, SZR-72 and SZR-104 (**Figures 2B, C**). All the components of the ROFA score showed significantly higher values in the saline-treated septic animals than in the sham-operated animals (**Supplementary Table S2**). Although the PaO_2_/FiO_2_ ratio and plasma levels of ALT and urea were similar to those in the sham-operated animals in all the treated groups, we highlight that SZR-72 was able to significantly reduce the liver damage caused by sepsis. Despite this, these treatments did not influence the overall ROFA score.

#### Changes in Blood–Brain Barrier Permeability and Brain Damage

Sepsis caused a significantly higher wet tissue/body weight ratio in the brain regions studied as compared with those in the sham-operated animals. However, these local manifestations of brain edema reached a lower extent in the hippocampus in all three of the treated septic groups and in the cerebellum in the septic groups treated with SZR-72 and SZR-104 (**Supplementary Figure S2**).

**Figure 3:**
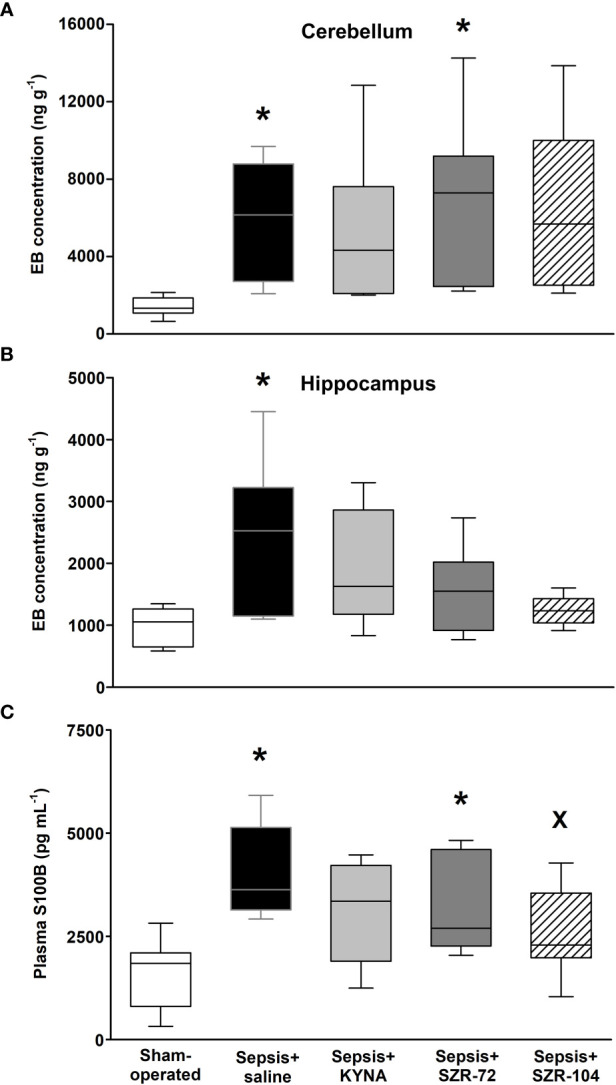
Blood–brain barrier permeability detected by the tissue levels of Evans Blue (EB) in the cerebellum **(A)** and hippocampus **(B)** and changes in plasma S100B levels **(C)** in a sham-operated group and in different sepsis groups treated with saline, KYNA, SZR-72 and SZR-104. The plots demonstrate the median (horizontal line in the box) and the 25^th^ (lower whisker) and 75^th^ (upper whisker) percentiles. Kruskal–Wallis test, Dunn’s *post-hoc* test; *P < 0.05 *vs*. sham-operated group; ^x^ *P* < 0.05 *vs*. sepsis+saline group.

Sepsis induced a significant increase in BBB permeability in the hippocampus and cerebellum as detected by the Evans Blue method (**Figures 3A, B**), which was not substantially influenced by any of the treatments in the cerebellum (**Figure 3A**). In the case of the hippocampus, EB extravasation reached a similar extent to that in the sham-operated group in all three of the treated septic groups (**Figure 3B**).

The plasma level of S100B was used as a marker of brain damage and an indirect measure of BBB disruption. Significantly higher S100B levels were observed in the saline- and SZR-72-treated septic groups as compared to those in the sham-operated animals, whereas this parameter was markedly lower in response to SZR-104 treatment than that in the saline-treated septic group (**Figure 3C**).

#### Changes in Markers of NET Formation

**Figure 4:**
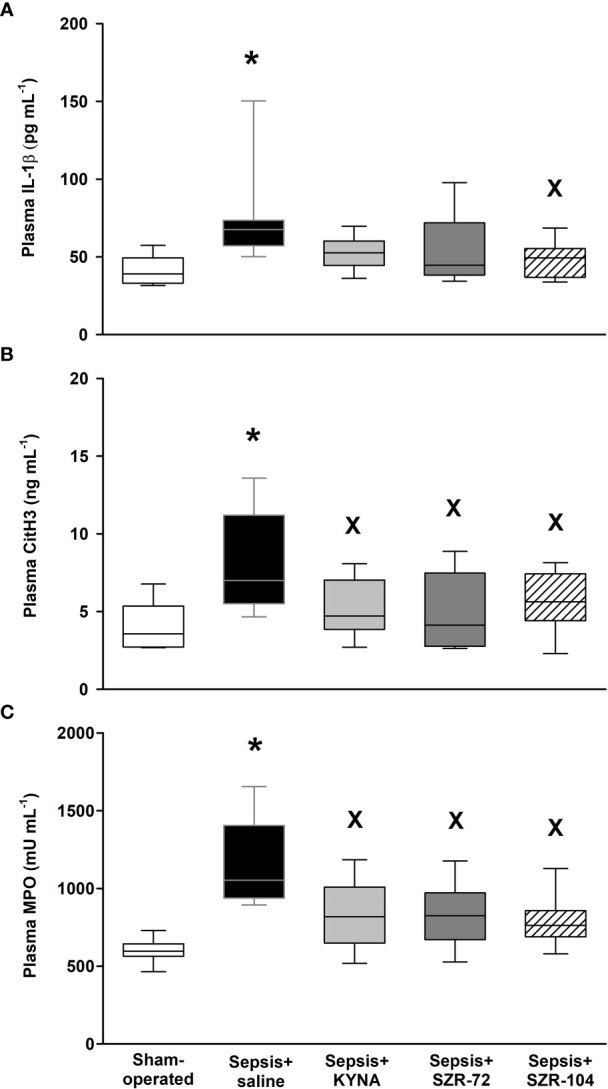
Plasma IL-1β **(A)**, citrullinated histone H3 (CitH3, **B**) and myeloperoxidase levels (MPO, **C**) in a sham-operated group and in different sepsis groups treated with saline, KYNA, SZR-72 and SZR-104. The plots demonstrate the median (horizontal line in the box) and the 25^th^ (lower whisker) and 75^th^ (upper whisker) percentiles. **(A)** Kruskal–Wallis test, Dunn’s post-hoc test; **(B, C)** One-way ANOVA, Holm–Sidak *post-hoc* test; *P < 0.05 *vs*. sham-operated group; ^x^ *P* < 0.05 *vs*. sepsis+saline group.

Plasma levels of IL-1β (an inducer of NETs) and CitH3 and MPO (the latter two being components of NETs formation) showed significantly higher plasma levels in the saline-treated septic groups than in any of the other groups (**Figures 4A–C**). Furthermore, no differences from the sham-operated groups were detected in any of the treated septic groups in these parameters. As compared to the values of the saline-treated sepsis groups, all the treatments resulted in significantly lower CitH3 and MPO values (**Figures 4B, C**), whereas IL-1β was lower only in response to SZR-104 treatment (**Figure 4A**).

**Figure 5:**
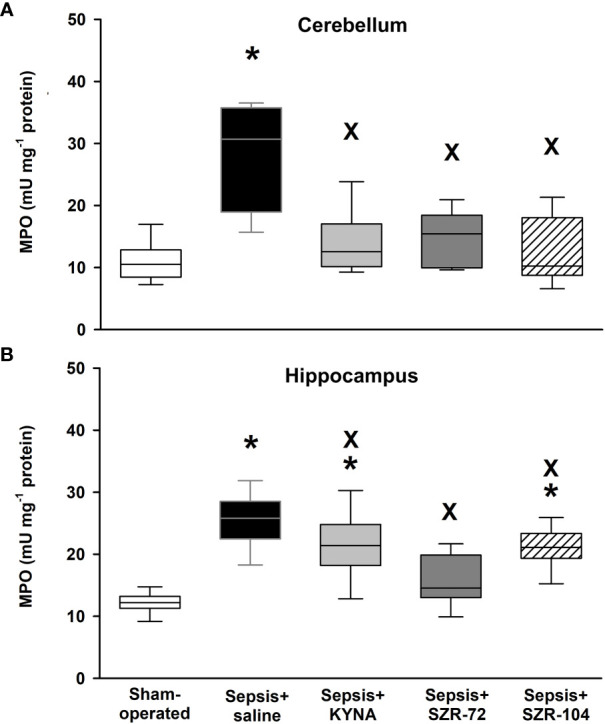
Cerebellar **(A)** and hippocampal **(B)** levels of myeloperoxidase (MPO) in the sham-operated group and in the different sepsis groups treated with saline, KYNA, SZR-72 and SZR-104. The plots demonstrate the median (horizontal line in the box) and the 25^th^ (lower whisker) and 75^th^ (upper whisker) percentiles. Kruskal–Wallis test, Dunn’s *post-hoc* test; *P < 0.05 *vs*. sham-operated group; ^x^ *P* < 0.05 *vs*. sepsis+saline group.

Sepsis also caused significant increases in tissue MPO content (an indicator of neutrophil leukocyte infiltration) in both hippocampal and cerebellar tissues (**Figures 5A, B**). These elevations were not present in the cerebellum in any of the treated groups, and all three treatments resulted in significantly lower tissue MPO values than untreated sepsis. In the hippocampus, higher tissue MPO values were also present in the KYNA and SZR-104-treated animals than those of the sham operation, but also significantly lower levels were evident in response to all three treatments than those after the saline treatment (**Figure 5B**).

#### Changes in Cerebral Mitochondrial Functions

**Figure 6:**
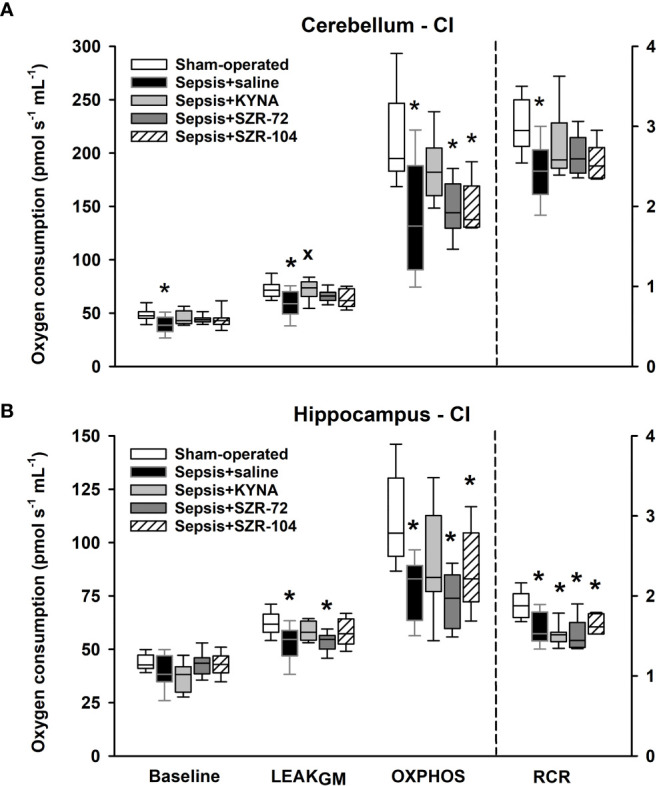
Complex I-linked mitochondrial oxygen consumption in the cerebellum **(A)** and the hippocampus **(B)** in the sham-operated group and in the different sepsis groups treated with saline, KYNA, SZR-72 and SZR-104. The plots demonstrate the median (horizontal line in the box) and the 25^th^ (lower whisker) and 75^th^ (upper whisker) percentiles. One-way ANOVA, Holm–Sidak *post-hoc* test; *P < 0.05 *vs*. sham-operated group; ^x^ *P* < 0.05 *vs*. sepsis+saline group.

As a result of sepsis, both cerebellar and hippocampal complex I-linked respirations were significantly reduced, as indicated by decreased oxidation of complex I-linked substrates (LEAK_GM_), ADP-stimulated respiration (OXPHOS I) and respiratory acceptor control ratios (RCR I; **Figures 6A, B**). Neither KYNA nor KYNA analogues restored the sepsis-induced decrease in complex I-linked OXPHOS capacity, and RCR values remained close to the septic values. However, complex I-supported LEAK respiration (LEAK_GM_) was slightly, but significantly increased following KYNA therapy in the cerebellum.

**Figure 7:**
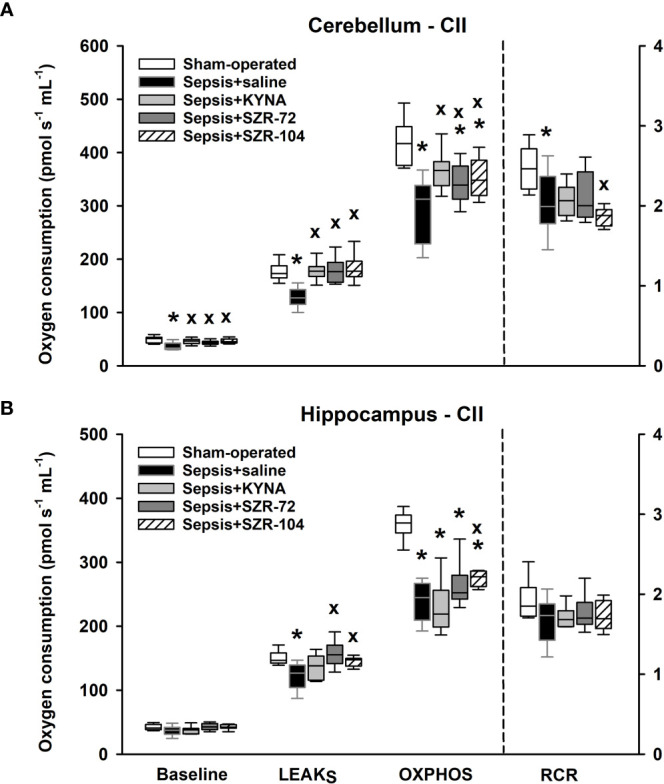
Complex II-linked mitochondrial oxygen consumption in the cerebellum **(A)** and the hippocampus **(B)** in the sham-operated group and in the different sepsis groups treated with saline, KYNA, SZR-72 and SZR-104. The plots demonstrate the median (horizontal line in the box) and the 25^th^ (lower whisker) and 75^th^ (upper whisker) percentiles. One-way ANOVA, Holm–Sidak *post-hoc* test; *P < 0.05 *vs*. sham-operated group; ^x^ *P* < 0.05 *vs*. sepsis+saline group.

Similarly, reduced complex II-dependent respirations were found following the septic insult, and there was a significant decrease in complex II-linked substrate oxidation (LEAK_S_) and OXPHOS II capacity in the hippocampal and cerebellar regions (**Figures 7A, B**). Remarkably, the treatments with KYNA, SZR-72 and SZR-104 improved LEAK_S_ and OXPHOS II in the cerebellum. Among the three compounds tested, only SZR-104 partially restored complex II-linked OXPHOS in the hippocampus.

## Discussion

The present study demonstrated the efficacy of KYNA and KYNA analogue-based treatments in reducing BBB injury and brain mitochondrial dysfunction during the early phases of experimental intraabdominal sepsis. These changes may occur due to NET-associated BBB injury. In this rodent model, proper analgesia, fluid resuscitation and sequential assessment of organ failure was performed according to the most recent recommendations (29). BBB change and brain injury were associated with local neutrophil infiltration, which likely coincided with systemic NET formation. In this scenario, KYNA and its synthetic analogues only influenced a few components of the ROFA score but exerted marked effects on plasma and the cerebral tissue components of NET formation. Further, the treatments significantly influenced sepsis-induced BBB permeability changes and mitochondrial respiration in the cerebellar and hippocampal regions.

Infiltration of immune cells into the CNS following BBB injury causes myelin degradation and axonal damage (4), thus underlining the importance of modulating BBB permeability during systemic inflammation. In our model, S100B served not only as a marker of astrocyte-derived brain injury but also as an indicator of early BBB damage during sepsis (8). In this model, a diffuse BBB dysfunction can be presumed because EB accumulation (a good indicator of macromolecular passage through the BBB), occurred in both the hippocampal and cerebellar regions. KYNA and KYNA analogues differentially affected these changes due to their different BBB penetration abilities. Unlike both synthetic analogues, KYNA poorly penetrates the BBB under normal physiological conditions (27, 28). However, we cannot rule out the possibility that BBB injury favored the penetration of all the compounds into the CNS at the time of the treatments (16h and 22h of sepsis). Both KYNA and SZR-104 significantly reduced (the latter more effectively) the sepsis-induced increase in S100B plasma levels. Recently, a link between NMDA-R and organization of the cytoskeleton in regulating BBB permeability causing reduced S100B release (32) and a shrinkage of brain endothelial cells *via* the cytoskeletal reorganization (*via* a NMDA-R triggered Rho-associated and a protein kinase-dependent phosphorylation of the myosin light chain) were also described (33). Therefore, the positive effect of SZR-104 on BBB integrity may also occur through the inhibition of NMDA-R-dependent signaling pathways (28).

A link between BBB damage and intravascular/intraparenchymal NET formation in the CNS has already been suggested (34), and reduced BBB damage as a consequence of neutrophil depletion has also been demonstrated (9). Since KYNA and KYNA analogues modulated NET components and inhibited neutrophil accumulation (and their potential-tissue damaging effects related to MPO and NADPH oxidase) in the brain, a causal relationship between ameliorated BBB function and attenuated NETosis is very likely. This assumption was supported by the strong correlation (Spearman coefficient; r=0.806; *P*<0.001) found between plasma CitH3 and plasma S100B levels in septic rats (**Supplementary Figure S3**).

NET formation/NETosis is a systemic phenomenon which plays a role in the pathogenesis of sepsis-associated organ dysfunction (35). Although numerous variable components of NETs have been identified, some structural elements of NETs are consistent and comprehensive biomarkers (13). Levels of many circulating NET markers (including plasma MPO, CitH3, circulating cell-free DNA-histone complexes) and NET inducers (IL-1β and interleukin-8; IL-8) increase under various inflammatory conditions (36, 37). Among these, CitH3 is highly specific to NET formation, and it was also suggested as a therapeutic target in experimental models of sepsis (38, 39). CitH3, together with its generating enzymes, peptidylarginine deiminases, was implicated in accelerating thrombotic processes (40), and their levels correlated with SOFA scores, disease severity and ICU mortality in septic shock patients (41, 42). In our study, apart from elevations in CitH3 plasma levels, plasma levels of the NET-inducer IL-1β and the neutrophil-derived MPO in plasma and brain tissues were also markedly increased in septic rats. The sepsis-induced increases in MPO and CitH3 levels in plasma were significantly reduced by KYNA and its analogues, and IL-1β plasma levels were also positively influenced by SZR-104.

The effects of KYNA and its analogues on neutrophil-dependent NET markers in the periphery and the CNS were first examined in our study. The more detailed mechanism of action calls for further investigations, but a concurrently reduced release of pro-inflammatory cytokines (tumor necrosis factor alpha (TNF-α); high mobility group box 1 (HMGB1) and IL-1β) cannot be ruled out, as shown in other studies (43–45). Previously, reduced tissue MPO and plasma pro-inflammatory markers (TNF-α, IL-6) were also demonstrated in response to KYNA and SZR-72 treatments in experimental colitis (24). Activated platelets induce extracellular trap formation *via* HMGB1 (46), and the role of HMGB1 in facilitating NETosis and mediating brain injury has also been demonstrated (47). An inhibitory effect of KYNA and SZR-72 on human mononuclear cell-derived TNF-α and HMGB1 production represents another pathway for the anti-inflammatory action of these compounds (48). In addition, IL-1β activation has been shown to drop following pretreatment with SZR-72 in a rat model of Complete Freund’s Adjuvant-induced dural inflammation (45). It is therefore plausible that KYNA and its derivatives inhibit NET formation during sepsis through the inhibition of TNF-α and HMGB1 release and IL-1β activation.

Crosstalk between mitochondria and NETosis has recently been emphasized. Vorobjeva et al. demonstrated that the calcium (Ca^2+^)-triggered opening of mitochondrial permeability transition pores (mPTPs) stimulated mitochondrial ROS production (mtROS), which in turn activated NET formation independently of NADPH oxidase and MPO in neutrophil leukocytes (49). On the other hand, NETs cause a reduction in the capillary–mitochondrial oxygen gradient by enhancing the formation of occluding thrombi (40), thereby impairing mitochondrial respiration. As regards organelle function, imbalance in mitochondrial homeostasis has previously been demonstrated in the liver after sepsis, and it was effectively reduced by KYNA and SZR-72 administration (17).

Our study has also shown that brain mitochondria are affected early after the septic insult, with a decrease in substrate (LEAK_GM_ and LEAK_S_) and ADP-activated respiration (OXPHOS) in cerebellar and hippocampal samples. These findings support previous reports on a decrease in mitochondrial activity (complex I) and enhanced ROS production in different brain regions following CLP-induced sepsis (16). One regulatory mechanism of OXPHOS machinery can be mediated through phosphorylation/dephosphorylation of respiratory chain proteins executed by opposing actions of tyrosine kinase Src and protein tyrosine phosphatase 1B (PTP1B). Sepsis or LPS stimuli initiate a pathologic imbalance between Src/PTP1B activities (decreased Src and increased PTP1B) that can ultimately reduce ATP synthesis in the brain (3, 50). Moreover, energy metabolism can also be reprogrammed from OXPHOS to aerobic glycolysis in microglial cells, resulting in less net gain energy production per glucose (15).

In our experiments, KYNA and KYNA derivative-based therapies ameliorated complex II-linked OXPHOS capacities and substrate-activated respiration in cerebellum samples. This finding is also consistent with other studies, in which KYNA improved various mitochondrial parameters, such as complex II activity, mitochondrial mass and membrane potential, antioxidant enzyme levels, and ROS against quinolinic acid-induced neuronal injury (51). There is also evidence that KYNA acting on G protein-coupled receptor GPR35 regulates adipocyte energy homeostasis *via* stimulation of lipid metabolism and mitochondrial respiration (52). Additionally, NMDA-Rs were shown to be present in the inner mitochondrial membrane (53), where they may play a regulatory role in Ca^2+^ transport. Inhibitory action exhibited by KYNA or its synthetic analogues on NMDA-R may reduce (I) receptor-driven Ca^2+^ influx, (II) mitochondrial Ca^2+^ overload and (III) release of apoptosis inducer cytochrome c (54). Although KYNA-mediated cell- and mito-protective properties were identified in models of various diseases, KYNA itself did not affect the bioenergetic function in the normal brain and normal liver mitochondria (55).

The present study was first to describe the mitochondrial effects of SZR-72 and SZR-104 in the CNS. Although only SZR-104 ameliorated complex II-linked OXPHOS in the hippocampus, all the treatments enhanced complex II-linked OXPHOS in the cerebellum without affecting complex I respiration. These differences may arise from the different membrane localization of complexes I and II. Besides, complex I is potentially more vulnerable to neuronal injury than other ETS components, and the level of pyridine nucleotide and other cofactors is also reduced during sepsis. There is also evidence that the hippocampus is more susceptible to insults, such as ischemia, anoxia, inflammation and sepsis (56). Therefore, a functional difference in distinct brain regions, particularly after BBB injury, cannot be ruled out. The mechanism by which SZR-72 and SZR-104 preserve brain mitochondrial function is not yet known with certainty; however, the microcirculatory improvement can be mediated with different receptors (NMDA-R and GPR35, respectively) (17). Better tissue oxygenation along the capillary–mitochondrial oxygen gradient may ameliorate oxygen consumption and subsequently results in better energy production in the organelle (26).

Our study has limitations as well. Firstly, the observation period was relatively short; detection of other endpoints (mortality or the cognitive component) would thus be needed in longer follow-up studies. Only PAD-dependent NET formation was addressed and plasma levels of CitH3 (and not DNA-CitH3 complexes) were determined in our study, and the number of inflammatory mediators (and NET inducers) examined was also limited. Furthermore, the current design did not allow us to investigate whether changes in BBB permeability are transient or irreversible or involve paracellular and/or transcellular pathways. The methodology applied did not allow for an assessment of any causal relationship between NET formation and BBB disintegration. Likewise, the effects of the ketamine-containing anesthetic agents cannot be disregarded. It should also be added that since antibiotics affect mitochondrial functions, this confounding option was purposefully omitted from the protocol (57).

**Figure 8:**
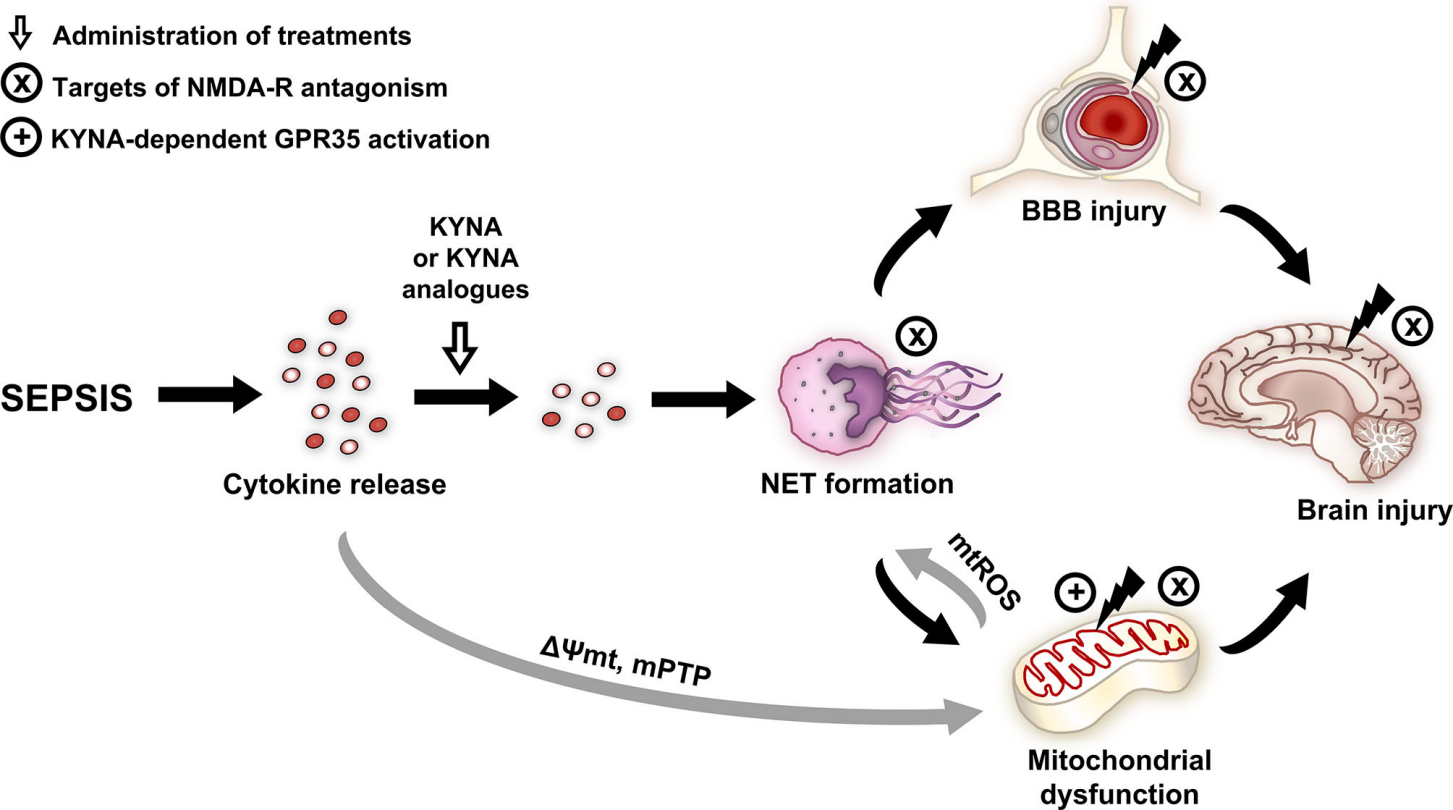
Effects and potential targets of KYNA and its analogues. NMDA and GPR35 receptor-dependent mechanisms are highlighted. Gray arrows indicate the literature-based mechanisms, and black arrows indicate the mechanisms examined in the study. BBB, blood–brain barrier; GPR35, G protein-coupled receptor 35; KYNA, kynurenic acid; mPTP, mitochondrial permeability transition pore; mtROS, mitochondrial reactive oxygen species; NET, neutrophil extracellular trap; NMDA-R, N-methyl-D-aspartate receptor; ΔΨmt, mitochondrial membrane potential.

In conclusion, KYNA and its analogues on NET-associated markers were first examined, and the compounds significantly attenuated sepsis-induced leukocyte activation and alleviated cerebral mitochondrial dysfunction. These compounds, either *via* inhibition NMDA-R or NET formation, may influence BBB permeability and mitochondrial damage, thereby reducing sepsis-related brain injury (**Figure 8**). Alternatively, KYNA- and analogue-based treatments may also affect tissue oxygenation, and, as a consequence, they may improve mitochondrial respiration. Our results suggest that KYNA or synthetic derivatives, particularly SZR-104, might be applicable as a supportive therapy in the treatment of the CNS-linked consequences of sepsis.

## Data Availability Statement

The raw data supporting the conclusions of this article will be made available by the authors, without undue reservation.

## Ethics Statement

The animal study was reviewed and approved by National Scientific Ethical Committee on Animal Experimentation under license number V/175/2018.

## Author Contributions

MP, AN, AR, LJ, ST, LG, BC, RG, and DD performed the *in vivo* experiments, AN, LJ, KG, and ST carried out the biochemical measurements. MP, JK, LJ, and AS wrote the manuscript. AN, AR and ST prepared the figures. FF, BL, and IS contributed to new KYNA analog. AS, JK, MB, and LV supervised and edited the manuscript. All authors contributed to the article and approved the submitted version.

## Funding

Sources of funding: NKFIH K116689, GINOP-2.3.2-15-2016-00034 and University of Szeged Open Access Fund (Grant number: 5312). This research was conducted with the support of the Szeged Scientists Academy under the sponsorship of the Hungarian Ministry of Human Capacities (EMMI: 13725-2/2018/INTFIN).

## Acknowledgments

We appreciate the excellent technical assistance from Csilla Mester and Krisztina Lívia Kovács. This work is dedicated to the memory of Prof. Ferenc Fülöp.

## Conflict of Interest

The authors declare that the research was conducted in the absence of any commercial or financial relationships that could be construed as a potential conflict of interest.

## Publisher’s Note

All claims expressed in this article are solely those of the authors and do not necessarily represent those of their affiliated organizations, or those of the publisher, the editors and the reviewers. Any product that may be evaluated in this article, or claim that may be made by its manufacturer, is not guaranteed or endorsed by the publisher.

## Supplementary Material

Supplementary Figure 1 Animal sickness assessment score in the sham-operated group and in the different sepsis groups treated with saline, KYNA, SZR-72 and SZR-104 taken 16h after sepsis induction. The plots demonstrate the median (horizontal line in the box) and the 25^th^ (lower whisker) and 75^th^ (upper whisker) percentiles. Kruskal–Wallis test, Dunn’s *post-hoc* test; **P* < 0.05 *vs*. sham-operated group.

Supplementary Figure 2 Cerebellar **(A)** and hippocampal **(B)** wet weight/body weight ratio in the sham-operated group and in the different sepsis groups treated with saline, KYNA, SZR-72 and SZR-104. The plots demonstrate the median (horizontal line in the box) and the 25^th^ (lower whisker) and 75^th^ (upper whisker) percentiles. Kruskal–Wallis test, Dunn’s *post-hoc* test; **P* < 0.05 *vs*. sham-operated group.

Supplementary Figure 3 Correlation between plasma citrullinated histone H3 (CitH3) and plasma S100B values in septic rats. Spearman’s correlation coefficient r values and (null hypothesis-related) *P* values are provided, regression line and 95% confidence interval are indicated.

## References

[1] Singer M Deutschman CS Seymour CW Shankar-Hari M Annane D Bauer M. The Third International Consensus Definitions for Sepsis and Septic Shock (Sepsis-3). JAMA (2016) 315:801–10. 10.1001/jama.2016.0287 PMC4968574 26903338

[2] Czempik PF Pluta MP Krzych ŁJ. Sepsis-Associated Brain Dysfunction: A Review of Current Literature. Int J Environ Res Public Health (2020) 17:5852. 10.3390/ijerph17165852 PMC7460246 32806705

[3] Lyu J Zheng G Chen Z Wang B Tao S Xiang D. Sepsis-Induced Brain Mitochondrial Dysfunction is Associated With Altered Mitochondrial Src and PTP1B Levels. Brain Res (2015) 1620:130–8. 10.1016/j.brainres.2015.04.062 25998537

[4] Sonneville R Verdonk F Rauturier C Klein IF Wolff M Annane D. Understanding Brain Dysfunction in Sepsis. Ann Intensive Care (2013) 3:15. 10.1186/2110-5820-3-15 23718252 PMC3673822

[5] Papadopoulos MC Lamb FJ Moss RF Davies DC Tighe D Bennett ED. Faecal Peritonitis Causes Oedema and Neuronal Injury in Pig Cerebral Cortex. Clin Sci (1999) 96:461–6. 10.1042/CS19980327 10209077

[6] Davies DC. Blood-Brain Barrier Breakdown in Septic Encephalopathy and Brain Tumours. J Anat (2002) 200:639–46. 10.1046/j.1469-7580.2002.00065.x PMC1570752 12162731

[7] Ari I Kafa IM Kurt MA. Perimicrovascular Edema in the Frontal Cortex in a Rat Model of Intraperitoneal Sepsis. Exp Neurol (2006) 198:242–9. 10.1016/j.expneurol.2005.12.001 16423349

[8] Aslankoc R Savran M Ozmen O Asci S. Hippocampus and Cerebellum Damage in Sepsis Induced by Lipopolysaccharide in Aged Rats - Pregabalin can Prevent Damage. BioMed Pharmacother (2018) 108:1384–92. 10.1016/j.biopha.2018.09.162 30372841

[9] Moxon-Emre I Schlichter LC. Neutrophil Depletion Reduces Blood-Brain Barrier Breakdown, Axon Injury, and Inflammation After Intracerebral Hemorrhage. J Neuropathol Exp Neurol (2011) 70:218–35. 10.1097/NEN.0b013e31820d94a5 21293296

[10] Brinkmann V Reichard U Goosmann C Fauler B Uhlemann Y Weiss DS. Neutrophil Extracellular Traps Kill Bacteria. Science (2004) 303:1532–5. 10.1126/science.1092385 15001782

[11] Manda-Handzlik A Demkow U. The Brain Entangled: The Contribution of Neutrophil Extracellular Traps to the Diseases of the Central Nervous System. Cells (2019) 8:1477. 10.3390/cells8121477 PMC6953104 31766346

[12] Yipp BG Kubes P. Netosis: How Vital is it? Blood (2013) 122:2784–94. 10.1182/blood-2013-04-457671 24009232

[13] Papayannopoulos V. Neutrophil Extracellular Traps in Immunity and Disease. Nat Rev Immunol (2018) 18:134–47. 10.1038/nri.2017.105 28990587

[14] Shen XF Cao K Jiang JP Guan WX Du JF. Neutrophil Dysregulation During Sepsis: An Overview and Update. J Cell Mol Med (2017) 21:1687–97. 10.1111/jcmm.13112 PMC5571534 28244690

[15] Baik SH Kang S Lee W Choi H Chung S Kim JI. A Breakdown in Metabolic Reprogramming Causes Microglia Dysfunction in Alzheimer’s Disease. Cell Metab (2019) 30:493–07.e6. 10.1016/j.cmet.2019.06.005 31257151

[16] Comim CM Rezin GT Scaini G Di-Pietro PB Cardoso MR Petronilho FC. Mitochondrial Respiratory Chain and Creatine Kinase Activities in Rat Brain After Sepsis Induced by Cecal Ligation and Perforation. Mitochondrion (2008) 8:313–8. 10.1016/j.mito.2008.07.002 18657632

[17] Juhász L Rutai A Fejes R Tallósy SP Poles MZ Szabó A. Divergent Effects of the N-Methyl-D-Aspartate Receptor Antagonist Kynurenic Acid and the Synthetic Analog SZR-72 on Microcirculatory and Mitochondrial Dysfunction in Experimental Sepsis. Front Med (2020) 7:566582. 10.3389/fmed.2020.566582 PMC7729001 33330526

[18] Nakahira K Hisata S Choi AM. The Roles of Mitochondrial Damage-Associated Molecular Patterns in Diseases. Antioxid Redox Signal (2015) 23:1329–50. 10.1089/ars.2015.6407 PMC4685486 26067258

[19] Kozlov AV Lancaster JR Jr Meszaros AT Weidinger A. Mitochondria-Meditated Pathways of Organ Failure Upon Inflammation. Redox Biol (2017) 13:170–81. 10.1016/j.redox.2017.05.017 PMC5458092 28578275

[20] Steckert AV Comim CM Mina F Mendonça BP Dominguini D Ferreira GK. Late Brain Alterations in Sepsis-Survivor Rats. Synapse (2013) 67:786–93. 10.1002/syn.21686 23740866

[21] Imamura Y Yoshikawa N Murkami Y Mitani S Matsumoto N Matsumoto H. Effect of Histone Acetylation on N-methyl-D-aspartate 2B Receptor Subunits and Interleukin-1 Receptors in Association With Nociception-Related Somatosensory Cortex Dysfunction in a Mouse Model of Sepsis. Shock (2016) 45(6):660–7. 10.1097/SHK.0000000000000547 26682951

[22] Zhang S Wang X Ai S Ouyang W Le Y Tong J. Sepsis-Induced Selective Loss of NMDA Receptors Modulates Hippocampal Neuropathology in Surviving Septic Mice. PloS One (2017) 12(11):e0188273. 10.1371/journal.pone.0188273 29176858 PMC5703474

[23] Vécsei L Szalárdy L Fülöp F Toldi J. Kynurenines in the CNS: Recent Advances and New Questions. Nat Rev Drug Discov (2013) 12:64–82. 10.1038/nrd3793 23237916

[24] Érces D Varga G Fazekas B Kovács T Tőkés T Tiszlavicz L. N-methyl-D-aspartate Receptor Antagonist Therapy Suppresses Colon Motility and Inflammatory Activation Six Days After the Onset of Experimental Colitis in Rats. Eur J Pharmacol (2012) 691:225–34. 10.1016/j.ejphar.2012.06.044 22796676

[25] Tanaka M Bohár Z Vécsei L. Are Kynurenines Accomplices or Principal Villains in Dementia? Maintenance of Kynurenine Metabolism. Molecules (2020) 25:564. 10.3390/molecules25030564 PMC7036975 32012948

[26] Rutai A Fejes R Juhász L Tallósy SP Poles MZ Földesi I. Endothelin A and B Receptors: Potential Targets for Microcirculatory-Mitochondrial Therapy in Experimental Sepsis. Shock (2020) 54:87–95. 10.1097/SHK.0000000000001414 31318833

[27] Fülöp F Szatmári I Vámos E Zádor D Toldi J Vécsei L. Syntheses, Transformations and Pharmaceutical Applications of Kynurenic Acid Derivatives. Curr Med Chem (2009) 16:4828–42. 10.2174/092986709789909602 19929784

[28] Molnár K Lőrinczi B Fazakas C Szatmári I Fülöp F Kmetykó N. Szr-104, a Novel Kynurenic Acid Analogue With High Permeability Through the Blood-Brain Barrier. Pharmaceutics (2021) 13:61. 10.3390/pharmaceutics13010061 33466551 PMC7824826

[29] Osuchowski MF Ayala A Bahrami S Bauer M Boros M Cavaillon JM. Minimum Quality Threshold in Pre-Clinical Sepsis Studies (Mqtipss): An International Expert Consensus Initiative for Improvement of Animal Modeling in Sepsis. Shock (2018) 50:377–80. 10.1097/SHK.0000000000001212 PMC6133201 30106875

[30] Margotti W Giustina AD de Souza Goldim MP Hubner M Cidreira T Denicol TL. Aging Influences in the Blood-Brain Barrier Permeability and Cerebral Oxidative Stress in Sepsis. Exp Gerontol (2020) 140:111063. 10.1016/j.exger.2020.111063 32827711

[31] Kuebler WM Abels C Schuerer L Goetz AE. Measurement of Neutrophil Content in Brain and Lung Tissue by a Modified Myeloperoxidase Assay. Int J Microcirc (1996) 16:89–97. 10.1056/NEJM199607183350313 8737712

[32] Neuhaus W Freidl M Szkokan P Berger M Wirth M Winkler J. Effects of NMDA Receptor Modulators on a Blood-Brain Barrier In Vitro Model. Brain Res (2011) 1394:49–61. 10.1016/j.brainres.2011.04.003 21549356

[33] Mehra A Guérit S Macrez R Gosselet F Sevin E Lebas H. Nonionotropic Action of Endothelial NMDA Receptors on Blood-Brain Barrier Permeability Via Rho/ROCK-mediated Phosphorylation of Myosin. J Neurosci (2020) 40:1778–87. 10.1523/JNEUROSCI.0969-19.2019 PMC7046331 31953371

[34] Zenaro E Pietronigro E Della Bianca V Piacentino G Marongiu L Budui S. Neutrophils Promote Alzheimer’s Disease-Like Pathology and Cognitive Decline Via LFA-1 Integrin. Nat Med (2015) 21:880–6. 10.1038/nm.3913 26214837

[35] Colón DF Wanderley CW Franchin M Silva CM Hiroki CH Castanheira FVS. Neutrophil Extracellular Traps (Nets) Exacerbate Severity of Infant Sepsis. Crit Care (2019) 23:113. 10.1186/s13054-019-2407-8 30961634 PMC6454713

[36] Schechter MC Buac K Adekambi T Cagle S Celli J Ray SM. Neutrophil Extracellular Trap (NET) Levels in Human Plasma are Associated With Active TB. PloS One (2017) 12:e0182587. 10.1371/journal.pone.0182587 28777804 PMC5544211

[37] Eilenberg W Zagrapan B Bleichert S Ibrahim N Knöbl V Brandau A. Histone Citrullination as a Novel Biomarker and Target to Inhibit Progression of Abdominal Aortic Aneurysms. Transl Res (2021) 233:32–46. 10.1016/j.trsl.2021.02.003. S1931-5244(21)00026-8. 33571683

[38] Wong SL Wagner DD. Peptidylarginine Deiminase 4: A Nuclear Button Triggering Neutrophil Extracellular Traps in Inflammatory Diseases and Aging. FASEB J (2018) 32:fj201800691R. 10.1096/fj.201800691R PMC6219837 29924943

[39] Deng Q Pan B Alam HB Liang Y Wu Z Liu B. Citrullinated Histone H3 as a Therapeutic Target for Endotoxic Shock in Mice. Front Immunol (2020) 10:2957. 10.3389/fimmu.2019.02957 31998291 PMC6962130

[40] Sorvillo N Mizurini DM Coxon C Martinod K Tilvawala R Cherpokova D. Plasma Peptidylarginine Deiminase Iv Promotes Vwf-Platelet String Formation and Accelerates Thrombosis After Vessel Injury. Circ Res (2019) 125:507–19. 10.1161/CIRCRESAHA.118.314571 PMC6697196 31248335

[41] Costa NA Gut AL Azevedo PS Polegato BF Magalhães ES Ishikawa LLW. Peptidylarginine Deiminase 4 Concentration, But Not PADI4 Polymorphisms, Is Associated With ICU Mortality in Septic Shock Patients. J Cell Mol Med (2018) 22:4732–7. 10.1111/jcmm.13717 PMC6156447 30044533

[42] Tian Y Russo RM Li Y Karmakar M Liu B Puskarich MA. Serum Citrullinated Histone H3 Concentrations Differentiate Patients With Septic Verses non-Septic Shock and Correlate With Disease Severity. Infection (2021) 49:83–93. 10.1007/s15010-020-01528-y 33000445 PMC7527151

[43] Mándi Y Endrész V Mosolygó T Burián K Lantos I Fülöp F. The Opposite Effects of Kynurenic Acid and Different Kynurenic Acid Analogs on Tumor Necrosis Factor-α (Tnf-α) Production and Tumor Necrosis Factor-Stimulated Gene-6 (TSG-6) Expression. Front Immunol (2019) 10:1406. 10.3389/fimmu.2019.01406 31316502 PMC6611419

[44] Moroni F Cozzi A Sili M Mannaioni G. Kynurenic Acid: A Metabolite With Multiple Actions and Multiple Targets in Brain and Periphery. J Neural Transm (2012) 119:133–9. 10.1007/s00702-011-0763-x 22215208

[45] Lukács M Warfvinge K Kruse LS Tajti J Fülöp F Toldi J. KYNA Analogue SZR72 Modifies CFA-induced Dural Inflammation- Regarding Expression of pERK1/2 and IL-1β in the Rat Trigeminal Ganglion. J Headache Pain (2016) 17(1):64. 10.1186/s10194-016-0654-5 27377707 PMC4932003

[46] Maugeri N Campana L Gavina M Covino C de Metrio M Panciroli C. Activated Platelets Present High Mobility Group Box 1 to Neutrophils, Inducing Autophagy and Promoting the Extrusion of Neutrophil Extracellular Traps. J Thromb Haemost (2014) 12:2074–88. 10.1111/jth.12710 25163512

[47] Kim SW Lee H Lee HK Kim ID Lee JK. Neutrophil Extracellular Trap Induced by HMGB1 Exacerbates Damages in the Ischemic Brain. Acta Neuropathol Commun (2019) 7:94. 10.1186/s40478-019-0747-x 31177989 PMC6556959

[48] Tiszlavicz Z Németh B Fülöp F Vécsei L Tápai K Ocsovszky I. Different Inhibitory Effects of Kynurenic Acid and a Novel Kynurenic Acid Analogue on Tumour Necrosis Factor-α (Tnf-α) Production by Mononuclear Cells, HMGB1 Production by Monocytes and HNP1-3 Secretion by Neutrophils. Naunyn Schmiedebergs Arch Pharmacol (2011) 383:447–55. 10.1007/s00210-011-0605-2 21336543

[49] Vorobjeva N Galkin I Pletjushkina O Golyshev S Zinovkin R Prikhodko A. Mitochondrial Permeability Transition Pore is Involved in Oxidative Burst and NETosis of Human Neutrophils. Biochim Biophys Acta Mol Basis Dis (2020) 1866:165664. 10.1016/j.bbadis.2020.165664 31926265

[50] Arachiche A Augereau O Decossas M Pertuiset C Gontier E Letellier T. Localization of PTP-1B, Shp-2, and Src Exclusively in Rat Brain Mitochondria and Functional Consequences. J Biol Chem (2008) 283:24406–11. 10.1074/jbc.M709217200 PMC3259839 18583343

[51] Ferreira FS Biasibetti-Brendler H Pierozan P Schmitz F Bertó CG Prezzi CA. Kynurenic Acid Restores Nrf2 Levels and Prevents Quinolinic Acid-Induced Toxicity in Rat Striatal Slices. Mol Neurobiol (2018) 55:8538–49. 10.1007/s12035-018-1003-2 29564809

[52] Agudelo LZ Ferreira DMS Cervenka I Bryzgalova G Dadvar S Jannig PR. Kynurenic Acid and Gpr35 Regulate Adipose Tissue Energy Homeostasis and Inflammation. Cell Metab (2018) 27:378–92.e5. 10.1016/j.cmet.2018.01.004 29414686

[53] Nesterov SV Skorobogatova YA Panteleeva AA Pavlik LL Mikheeva IB Yaguzhinsky LS. NMDA and GABA Receptor Presence in Rat Heart Mitochondria. Chem Biol Interact (2018) 291:40–6. 10.1016/j.cbi.2018.06.004 29883723

[54] Ma T Cheng Q Chen C Luo Z Feng D. Excessive Activation of NMDA Receptors in the Pathogenesis of Multiple Peripheral Organs Via Mitochondrial Dysfunction, Oxidative Stress, and Inflammation. SN Compr Clin Med (2020) 2:551–69. 10.1007/s42399-020-00298-w

[55] Baran H Staniek K Bertignol-Spörr M Attam M Kronsteiner C Kepplinger B. Effects of Various Kynurenine Metabolites on Respiratory Parameters of Rat Brain, Liver and Heart Mitochondria. Int J Tryptophan Res (2016) 9:17–29. 10.4137/IJTR.S37973 27226722 PMC4872644

[56] Yuan M Yan DY Xu FS Zhao YD Zhou Y Pan LF. Effects of Sepsis on Hippocampal Volume and Memory Function. World J Emerg Med (2020) 11:223–30. 10.5847/wjem.j.1920-8642.2020.04.004 PMC7517393 33014218

[57] Moullan N Mouchiroud L Wang X Ryu D Williams EG Mottis A. Tetracyclines Disturb Mitochondrial Function Across Eukaryotic Models: A Call for Caution in Biomedical Research. Cell Rep (2015) 10:1681–91. 10.1016/j.celrep.2015.02.034 PMC4565776 25772356

