# Supplementary figures and images for: Kynurenic Acid and Its Synthetic Derivatives Protect Against Sepsis-Associated Neutrophil Activation and Brain Mitochondrial Dysfunction in Rats
Source: Front Immunol. 2021 Aug 12;12:717157. doi: 10.3389/fimmu.2021.717157 (PMC8406694; doi:10.3389/fimmu.2021.717157)

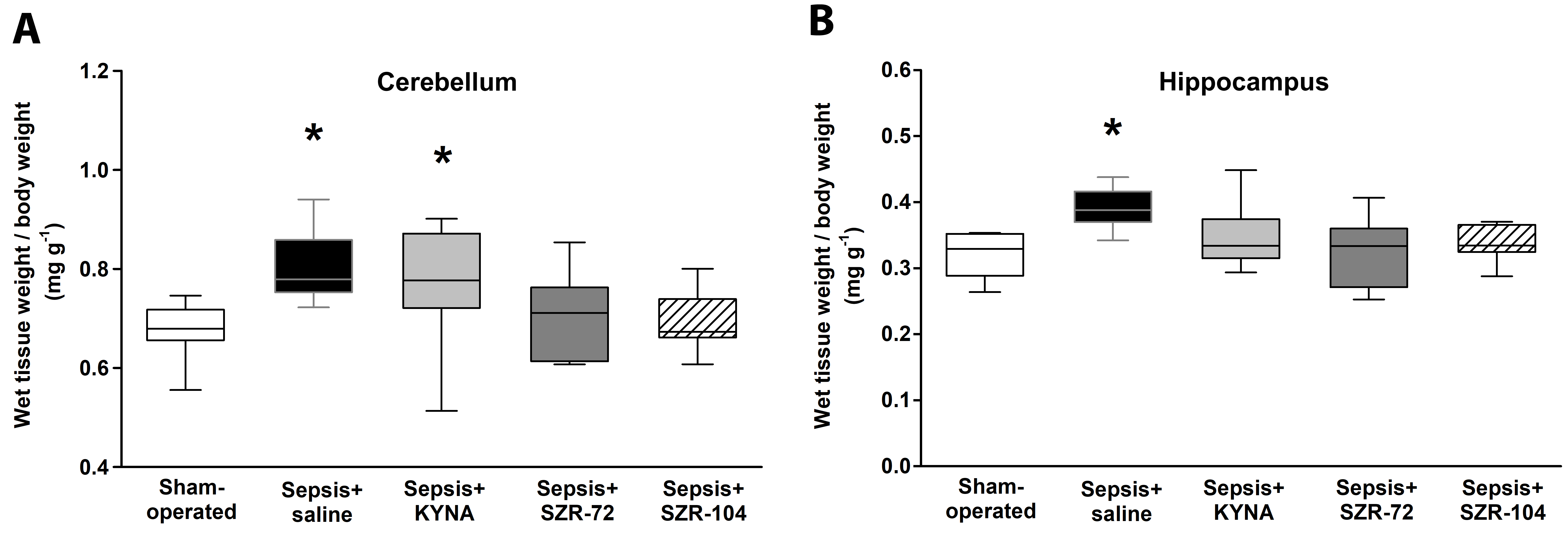

Supplement: Supplementary Figure 2 — Cerebellar (A) and hippocampal (B) wet weight/body weight ratio in the sham-operated group and in the different sepsis groups treated with saline, KYNA, SZR-72 and SZR-104. The plots demonstrate the median (horizontal line in the box) and the 25th (lower whisker) and 75th (upper whisker) percentiles. Kruskal–Wallis test, Dunn’s post-hoc test; *P < 0.05 vs. sham-operated group. [file Image_2.tif]

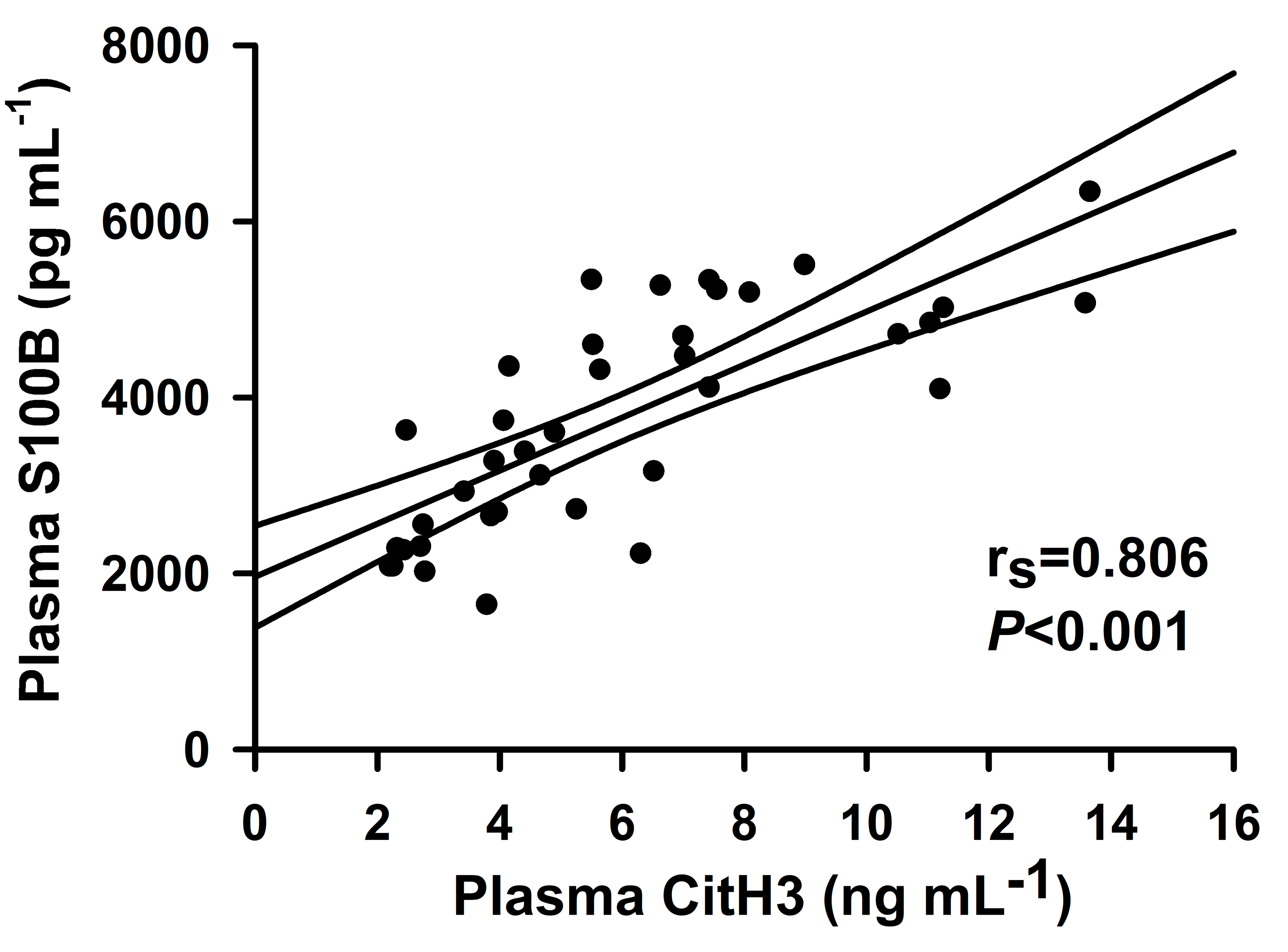

Supplement: Supplementary Figure 3 — Correlation between plasma citrullinated histone H3 (CitH3) and plasma S100B values in septic rats. Spearman’s correlation coefficient r values and (null hypothesis-related) P values are provided, regression line and 95% confidence interval are indicated. [file Image_3.tif]
