# Supplementary material for: Kynurenic Acid and Its Synthetic Derivatives Protect Against Sepsis-Associated Neutrophil Activation and Brain Mitochondrial Dysfunction in Rats
Source: Front Immunol. 2021 Aug 12;12:717157. doi: 10.3389/fimmu.2021.717157 (PMC8406694; doi:10.3389/fimmu.2021.717157)
Supplement: Supplementary file 4 [file Table_1.pdf]

Supplemental Table 1. Assessment scheme for the condition of the animals with rat sickness scores.

Parameters	Score	Observation
**Fur**	0	not altered
	1	piloerection, dirty fur
**Posture**	0	not altered
	1	altered weight distribution
	2	hunched back, tremors
**Mobility**	0	not altered
	1	slow/stiff movement when disturbed
	2	no movement when disturbed
**Alertness**	0	not altered
	1	decreased
	2	no reaction
**Weight**	0	+0-5-10 g
	1	-5-10 g
	2	-20 g
**Startle reflex**	0	normal
	1	slow
	E	no reaction
**Respiration**	0	not altered
	1	increased/decreased
**Body temperature**	0	not altered
	1	hypothermia/fever
