# Supplementary material for: Kynurenic Acid and Its Synthetic Derivatives Protect Against Sepsis-Associated Neutrophil Activation and Brain Mitochondrial Dysfunction in Rats
Source: Front Immunol. 2021 Aug 12;12:717157. doi: 10.3389/fimmu.2021.717157 (PMC8406694; doi:10.3389/fimmu.2021.717157)
Supplement: Supplementary file 5 [file Table_2.pdf]

Supplemental Table 2. Organ-specific changes in the different groups. Components and cumulative values of the rat-specific organ failure assessment (ROFA) score in the sham-operated, sepsis and treated sepsis groups.

ROFA Parameters	Groups				
	**Sham-operated**	**Sepsis + saline**	**Sepsis + KYNA**	**Sepsis + SZR-72**	**Sepsis + SZR-104**
**Lactate (mmol L^{-1})**	1.1 (1.1; 1.4)	**3.0 (2.3; 3.8)***	**2.5 (2.0; 3.4)***	**2.8 (2.4; 3.6)***	**2.9 (2.5; 3.4)***
**Mean arterial pressure (mm Hg)**	92 (84.8; 95.5)	**73.0 (57.0; 80.0)***	69.5 (63.0; 87.8)	**71.5 (58.3; 76.5)***	**67.0 (58.0; 74.5)***
**PaO_2 FiO_2^{-1} ratio**	393.3 (381.8; 442.9)	**315.7 (283.3; 369.3)***	367.6 (344.8; 408.6)	358.095 (293.1; 406.4)	388.1 (313.1; 451.9)
**Plasma ALT (U L^{-1})**	23.5 (13.3; 36.8)	**51.0 (42.0; 88.0)***	39.5 (32.0; 48.3)	**24.0 (21.0; 40.0)^{X}**	36.0 (29.5; 57.0)
**Plasma urea (mmol L^{-1})**	6.3 (5.9; 7.0)	**11.8 (7.8; 20.0)***	8.9 (6.2; 10.7)	6.4 (5.3; 17.2)	8.8 (7.2; 11.1)
**ROFA score**	1.5 (1.0; 2.0)	**6.0 (4.0; 7.8)***	**4.9 (4.0; 5.5)***	**4.5 (2.0; 7.0)***	**5.0 (3.0; 6.0)***

ALT: alanine aminotransferase. Data demonstrate the median and 25th and 75th percentiles. Comparison between groups was conducted with the Kruskal–Wallis test followed by Dunn's post-hoc test. $^{*}P<0.05$ vs. sham-operated groups (in boldface); $^{x}P<0.05$ vs. untreated sepsis group (in boldface).
